# Using a Large Language Model to Support Thematic Analysis of Patient Experiences in Chronic Illness Management: Comparative Qualitative Study

**DOI:** 10.2196/88677

**Published:** 2026-06-29

**Authors:** Sara Kivity, Yechiel Michael Barilan, Reut Noham, Mor Saban

**Affiliations:** 1 School of Medicine, Gray Faculty of Medical and Health Sciences Tel Aviv University Tel Aviv, Tel Aviv Israel; 2 Department of Industrial Engineering, Tel Aviv University Tel Aviv Israel; 3 Nursing Department, The Stanley Steyer School of Health Professions, Gray Faculty of Medical and Health Sciences Tel Aviv University Tel Aviv Israel

**Keywords:** chronic illness, disease management, qualitative research, patient experience, large language model

## Abstract

**Background:**

Qualitative health research often focuses on how patients experience and manage chronic illnesses, a topic that has been extensively studied in the literature. With the emergence of large language models (LLMs), such as Claude (Anthropic PBC) and ChatGPT (OpenAI), new opportunities are arising to support and scale the thematic analysis of narrative health data. However, their role and added value compared to traditional human-led approaches remain underexplored, particularly in complex clinical contexts such as multimorbidity.

**Objective:**

We aim to evaluate the methodological contribution of LLM-assisted analysis by examining its ability to replicate and extend established qualitative insights, in comparison with traditional thematic analysis.

**Methods:**

Semistructured interviews were conducted with 30 individuals living with two or more chronic illnesses. Transcripts were analyzed using both manual thematic coding and Claude 3.5 Sonnet. A structured comparison was conducted to identify shared and unique themes across the two approaches. The analysis examined thematic overlap, differences in subtheme identification, and variation in the level of detail between the methods.

**Results:**

Both approaches identified similar core themes related to the patient experience, including health care navigation and challenges, support systems and family dynamics, and emotional challenges and coping. Manual analysis produced more contextually detailed interpretations, while the LLM approach identified a larger number of subthemes. Each method also revealed distinct themes: the manual analysis included themes such as faith, caregiving roles, and a proactive mindset, whereas the LLM identified themes such as future planning and multiple health conditions. The findings show both similarities and differences between the two approaches. The LLM analysis also demonstrated efficiency in processing large volumes of qualitative data.

**Conclusions:**

A hybrid approach that integrates artificial intelligence–assisted and human-led thematic analysis can enhance both analytical depth and scalability. These findings support the use of LLMs as a complementary tool in qualitative research, while highlighting the importance of combining automated pattern detection with human interpretation.

## Introduction

Chronic illness management represents one of the most significant challenges in modern health care, requiring patients to navigate complex systems of care while integrating numerous health-related tasks into their daily lives [1,2]. Indeed, such management has evolved to include a broad spectrum of activities that patients must master and coordinate to the extent that they often develop what researchers term a “management career.” This refers to the dynamic process of learning, adapting, and integrating various health-related tasks into daily routines [3,4]. These include medication management, symptom monitoring, communication with health care providers, navigating insurance and health care systems, and addressing the psychological and social impact of their condition [2,3]. Moreover, patients often face competing demands from different conditions and must navigate conflicting treatment recommendations, requiring them to make complex decisions about prioritizing various aspects of their care [2,4]. In addition, studies show that patients often reconstruct their personal identity to incorporate their role as health managers, while simultaneously navigating changes in their social and professional relationships [5,6]. Thus, the impact of chronic illness management extends across multiple aspects of patients’ lives, constituting a dynamic “career” that goes beyond mere medical adherence [7]. Accordingly, research has identified several key factors that influence the management of chronic illnesses. These include social support networks, including family members, friends, and health care providers [1,2], patients’ self-efficacy and problem-solving skills, and the ability to navigate health care systems and coordinate care efficiently [8,9].

Qualitative studies are commonly used in exploring the chronic patient experience in disease management, as they provide rich and contextualized insights into the lived experiences of individuals [10]. However, manual thematic qualitative analysis, while valuable, faces several limitations: it is time-intensive and resource-demanding [11,12]. This approach presents challenges when applied to large datasets, as maintaining analytical depth across numerous interviews requires substantial effort [13,14]. While human interpretation is central to qualitative research and contributes to its richness, the scalability of manual analysis is limited, particularly in team-based settings, where variations in coding and interpretation may affect consistency [15,16]. In recent years, the emergence of large language models (LLMs), such as ChatGPT or Claude, has introduced new possibilities for qualitative research [1,17,18]. This scalability is a key advantage over traditional qualitative analysis, which is often limited by the time-consuming nature of manual coding and thematic interpretation [19,20]. LLMs may offer a different analytical lens to qualitative data analysis by identifying patterns across large textual datasets and generating thematic insights. Their ability to support reflexivity and reveal alternative interpretive frames makes them increasingly relevant in health research involving complex patient narratives. Rather than replacing the researcher’s interpretive role, the LLM is used to support reflexivity by surfacing alternative thematic groupings that could either challenge or complement the researchers’ assumptions [20,21]. In addition, as such models are often trained on multilingual and culturally diverse datasets, they may help uncover insights that traditional approaches might overlook, particularly those obscured by language or cultural framing [21,22].

To examine this potential in a meaningful way, it is useful to apply LLM-assisted analysis in a well-studied domain. Chronic illness experience has been widely studied in qualitative research, and many common themes are already known. This makes it a good domain for this study, as it allows us to compare the results of LLM-assisted analysis with existing knowledge. This study aimed to evaluate the contribution of LLM-assisted analysis compared to traditional thematic analysis by examining its ability to replicate and extend known qualitative insights in chronic illness management.

## Methods

### Study Design and Participants

**Figure 1:**
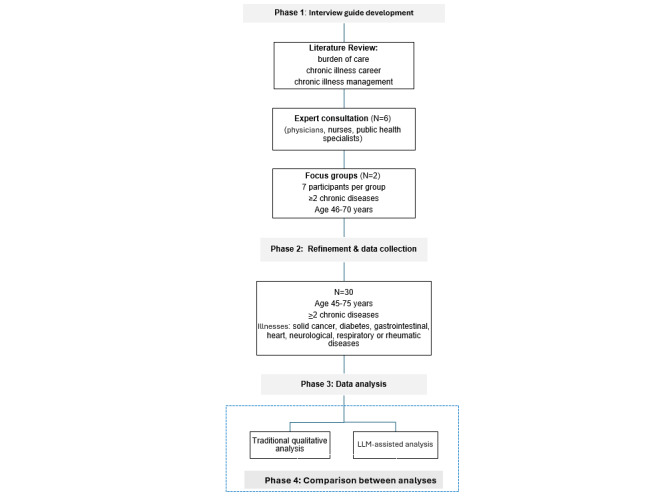
Overview of this study’s design and research procedure, including the phases of interview development, data collection, and parallel analysis using manual thematic analysis and LLM-assisted analysis, followed by a comparative evaluation. LLM: large language model.

This study used thematic analysis within an interpretivist framework, guided by the Standards for Reporting Qualitative Research [23,24]. To explore recurring patterns in participants’ experiences, semistructured interviews were conducted with individuals managing multiple chronic conditions, allowing for the identification of cross-cutting themes across diverse personal narratives. To investigate the concept of chronic illness management as a “career,” this study followed a multiphase qualitative approach designed to integrate both experiential depth and methodological comparison. The first phase involved the development of a semistructured interview guide, informed by literature review, expert consultation, and focus groups with patients. The second phase included refinement of the guide and data collection through interviews. In the third phase, interview transcripts were analyzed using two distinct methods: manual thematic analysis and artificial intelligence (AI)–assisted analysis using an LLM. The final phase focused on a comparative evaluation of the two analytical approaches, assessing similarities, differences, and unique contributions. This phased design allowed us to explore both patient experiences and the use of LLMs in qualitative analysis, as shown in Figure 1. This study followed an inductive analytical approach. While existing literature informed the development of the interview guide, no formal theoretical framework was systematically applied in either the manual or the LLM-assisted analysis.

### Procedure

#### Phase 1: Development of the Semistructured Interview Guide

A comprehensive literature review was conducted in January 2024, using the keywords: “burden of care,” “chronic illness career,” and “chronic illness management” to develop a conceptual framework and establish this study’s initial focus. Based on the findings, guides for semistructured interviews were designed and refined with input from a panel of experts with diverse backgrounds in health care and research. To further enhance this study, two focus groups were conducted with individuals who were not part of the primary study population. These focus groups were used to test the clarity, scope, and language of the interview questions, and to ensure that the guide addressed meaningful aspects of chronic illness management from a patient perspective, including the following: participants’ general background, the transition from health to illness, the “career” of managing chronic illness, balancing illness management with other life responsibilities, coping strategies, task prioritization, support systems, interactions with health care providers, and expectations for the future.

#### Phase 2: Refinement and Data Collection

This study was conducted over two months from February 2024 to March 2024. Participants were recruited through recommendations from their primary care physicians or other medical staff involved in their treatment. Referrals were based on clinical judgment and familiarity with the patients’ chronic conditions, functional status, and communication ability. Patients unable to engage in a coherent conversation were not referred. Each patient was individually approached, informed about this study, and provided written informed consent to participate. While thematic saturation was considered, a sample of 30 participants was purposefully selected to provide sufficient narrative depth for both manual and AI-assisted analyses. This study included 30 participants, aged 45 to 75 years, who were diagnosed with at least two of the following chronic illnesses: diabetes mellitus; congestive heart failure; chronic obstructive pulmonary disease; active oncological disease (or within one year of treatment); ischemic heart disease; chronic kidney disease (grade 3 or higher); inflammatory bowel disease (Crohn disease or ulcerative colitis); interstitial lung disease; epilepsy; multiple sclerosis; autoimmune conditions such as rheumatoid arthritis, psoriatic arthritis, systemic lupus erythematosus, or systemic sclerosis (scleroderma); or those undergoing anticoagulant treatment for at least three consecutive months (warfarin, heparin, or nonvitamin K antagonist oral anticoagulants). Requiring at least two conditions ensured a minimum level of complexity in participants’ health experiences. This broad inclusion reflects this study’s objective to capture cross-cutting experiences in multimorbidity, rather than focusing on a single condition. Participants underwent semistructured phone or videoconference interviews, chosen for their convenience. Interviews ranged in duration from 30 to 60 minutes, allowing participants to determine the depth and breadth of their narratives. The interview protocol included two main sections: (1) sociodemographic information: collecting data on gender, age, and primary illnesses; and (2) disease management: examining strategies, priority setting, task management, quality of life, coping techniques, and support systems. All data were manually transcribed to ensure accuracy.

#### Phase 3: Data Analysis

The data analysis involved two separate approaches: traditional qualitative methods and AI-assisted techniques, followed by a comparative evaluation to assess their respective insights and unique contributions.

In the traditional analysis, interview data were analyzed using an inductive thematic analysis approach, involving iterative coding, theme development, and refinement to identify key patterns across participants’ experiences [24]. This analysis was not theory-driven, and themes were generated inductively from the data. Initial codes were developed and refined through an iterative process. These codes were then categorized into broader themes that reflected the primary dimensions of the patients’ experiences. Shared themes were identified based on their recurrence across interviews and their conceptual relevance to this study’s aims. The manual analysis was conducted by a single researcher, who maintained detailed memos and engaged in reflective journaling to enhance transparency and reduce potential interpretive bias. This approach is consistent with qualitative research practices that emphasize reflexivity and transparency in the analytical process.

For the AI-assisted analysis, the LLM was implemented using the Claude.ai application programming interface with the following specifications: version: Claude 3.5 Sonnet (“claude-3.5 Sonnet-20240620”), temperature: 0, and maximum tokens: 8192. A custom Python script (version 3.10.11) was developed to enable the system to provide automated insights on key themes within the interviews. The LLM analysis was not theory-driven and did not include theory-based prompts. The interview transcripts were analyzed in full using a predefined and structured prompt that guided the model to identify key themes, categorize relevant passages, and extract illustrative quotes. The same prompt was applied to all transcripts to ensure consistency across analyses, and the resulting themes were organized for comparison with the manual analysis.

#### Phase 4: Comparative Analysis

The final phase focused on comparing the themes from the traditional qualitative analysis and the LLM-assisted analysis. A structured, side-by-side comparison of the themes generated by each method was conducted, using predefined criteria, including thematic overlap, identification of unique themes, and differences in the level of detail. Themes and subthemes were categorized as convergent when they reflected similar underlying concepts, even if expressed using different terminology, and as divergent when they represented distinct insights specific to one method. This comparison helped identify both the shared patterns and unique contributions of each approach. The comparative analysis was conducted by a single researcher, supported by systematic memo-based documentation.

### Ethical Considerations

This study was conducted in compliance with all relevant laws and institutional guidelines and was approved by the Tel Aviv University ethical review board. Formal ethical approval for the research was granted on February 13, 2023 (clinical trial number #0005962-1). Participation was voluntary, and informed consent was obtained from all participants before this study’s initiation. Participants were fully informed about the purpose, procedures, and potential risks of this study, and they retained the right to withdraw at any time without penalty. No financial or material compensation was provided. Confidentiality was ensured through anonymization of transcripts and secure data handling.

## Results

### Sample Characteristics

Table 1 summarizes the baseline characteristics of this study’s sample. The sample included 30 participants, 13 of whom were male, with an average age of 62.7 years (SD 7.3). The majority were either employed (n=19) or retired (n=10), with 7 of the 10 retirees attributing their retirement to medical reasons. The most common primary illnesses were diabetes (n=12) and rheumatic diseases (n=9). Most participants resided in the central region of the country (n=25), and 10 were the primary caregivers for a family member.

**Table 1:** Sociodemographic characteristics of this study’s sample (N=30).

		Overall	Missing (%)
Male, n (%)		13 (43.3)	0.0
**Employment status, n (%)**			3.3
	Employed	19 (63.3)	
	Retired	10 (33.3)	
	Unemployed	1 (3.4)	
Age (years), mean (SD)		62.7 (7.3)	0.0
Years since diagnosis, mean (SD)		11.7 (11.8)	10.0
**Primary illness, n (%)**			0.0
	Solid cancer	2 (6.7)	
	Diabetes mellitus	12 (40.0)	
	Gastrointestinal disease	1 (3.3)	
	Heart disease	3 (10.0)	
	Neurological diseases	2 (6.7)	
	Respiratory disease	1 (3.3)	
	Rheumatic diseases	9 (30.0)	
**Location, n (%)**			0.0
	Central	25 (83.3)	
Number of children, mean (SD)		2.7 (1.5)	3.3
Number of grandchildren, mean (SD)		3.3 (3.5)	20.0
Primary caregiver for a family member, n (%)		10 (33.3)	0.0

### Thematic Analysis

The traditional qualitative analysis identified six main themes (navigating daily life under chronic conditions, the role of support systems, health care and systemic challenges, emotional challenges and coping, self-management and adaptation, and sources of strength and motivation) as shown in Table 2, which presents the themes identified in the manual analysis along with illustrative participant quotes. The quotes presented in Table 2 provide contextual depth and support the interpretation of the identified themes. The LLM analysis also yielded six main themes (health management and adaptation, navigating health care systems, emotional challenges and coping, social and family dynamics, daily life and functional adjustments, and future planning and long-term concerns) as shown in Table 3, which presents the themes identified in the LLM-assisted analysis.

**Table 2:** Themes identified in the manual thematic analysis, with illustrative quotes from participants.

	Theme	Quote
1	Navigating daily life under chronic conditions	Patient 20: “…I had about a month filled with doing a million tests; they gave me again many tests: I had to go for a urine test, a lung test, and an eye test before I arrived at the eye department. The pulmonologist wanted me to do a lung X-ray, a CT scan, and blood tests.” Patient 4: “...I work at being chronically ill, because I have to keep going to doctors without knowing what I have. Yet again, the side effects. Each time a different side effect; sometimes it's the stomach pain, the leg pain,...”
2	The role of support systems	Patient 2: “…The community in the neighborhood where I live was very helpful and also brought food and made sure to help me at home. My sister cooked here and my mother. I learned to appreciate the power of family.” Patient 27: “I work around the clock, sometimes it involves 30, 40, 50 and even 60 hours continuously without sleep. That is, sleeping half an hour here, half an hour there in the car in between. One of the things that I think prevents me from deteriorating is the intensity of the work. Although it is said that this is what caused the disease to break out.”
3	Health care and systemic challenges	Patient 18: “When the problems with my legs started, I went to about four dermatologists in the community, and each of them gave me a completely different diagnosis. Until I got to a dermatologist at the hospital who took a biopsy and found my disease…” Patient 14: “When they started with the electronic prescriptions on the website, I suddenly found myself arriving at the pharmacy, waiting in line and then not having a prescription at all. It took me a while to figure out how things work... It was really one big headache...”
4	Emotional challenges and coping	Patient 6: “I added therapy to my life through gardening, work with animals. All kinds of niches that I didn't have the opportunity to do before the illness.. I try to protect my soul, try to satiate it with things that make me happy.” Patient 15: “...Look, even when the lupus nephritis broke out, that was also worrisome. I didn't have any symptoms but it was worrying.”
5	Self-management and adaptation	Patient 13: “…I don't give up on myself, once every four-six months, I write to the doctor and ask for periodic tests. I have to keep an eye on my health.” Patient 17: “The big difference started about three months ago when the glucose was suddenly high, I went to a dietitian, and I have already changed my eating habits. I need to do some sport but I don't persist with it.”
6	Sources of strength and motivation	Patient 15: “The doctors told me, ‘Well, you will not return to teaching’. And my husband said to the doctor, ‘Sorry but you don't know her, she will go back to teaching.’ I really didn't study the first semester; I rested at home. In the second semester, I returned to teaching.” Patient 9: “My strength comes from God. I say God help me with my hands up. And I know it will help with the disease; God is with you all the time. I am a religious woman, I believe in God.”

**Table 3:** Themes identified in the large language model–assisted analysis, with illustrative text excerpts generated from the model outputs.

	Theme	Quote
1	Health management and adaptation	Patient 14: “…With awareness over the years, it really became part of my management, this matter of sugar. I learned to accept it…” Patient 7: “I'm constantly going around to doctors a lot. Many times ... Sometimes if it's close to home I can go out carefully, very very slowly, like that.”
2	Navigating health care systems	Patient 20: “…I had about a month filled with doing a million tests… I had to go for a urine test, a lung test, and an eye test before I arrived at the eye department…I felt like I had been in the clinic for the whole month.” Patient 29: “…through all these months until I got to the department... because no doctor could help me or figure out what was wrong with me.”
3	Social and family dynamics	Patient 1: “Only in the last year when my hemoglobin dropped, my eldest daughter wouldn't let it go, she accompanied me, but really company? Let me be.” Patient 13: “Without such support from the environment it's impossible, I would have died. I know I would have died. I say with certainty that without my family, it's impossible, a lonely person doesn't survive this illness.”
4	Emotional challenges and coping	Patient 26: “I'm one of those who thought it wouldn't happen to me. Suddenly it happened. And yes, for me it was shocking, I'm so healthy and strong.” Patient 14: “When I saw him and he told me about Ozempic I had tears in my eyes, he asked me, 'Why are you crying?' and I told him, 'Listen, I feel like I failed.'”
5	Daily life and functional adjustments	Patient 2: “There are priorities, I worked all my life. The house isn't a museum but it needs to be clean and there's help once a week and I don't give up on that. And now with my eyes I'm not allowed to do many things so the helper comes twice a week.” Patient 27: “For example, during the disease attacks, I always take a bag packed with underwear and pants and everything...”
6	Future planning and long-term concerns	Patient 1: “Right now, currently, I need to protect the kidney. Because that's the most vulnerable thing, in terms of FMF as I understood. Although I understood it before, in the last year I understood even more, to protect them.” Patient 18: “Not infrequently, I think about the diseases that I have. That can come to expression…I don't want to say it but can shorten a person's life.”

**Figure 2:**
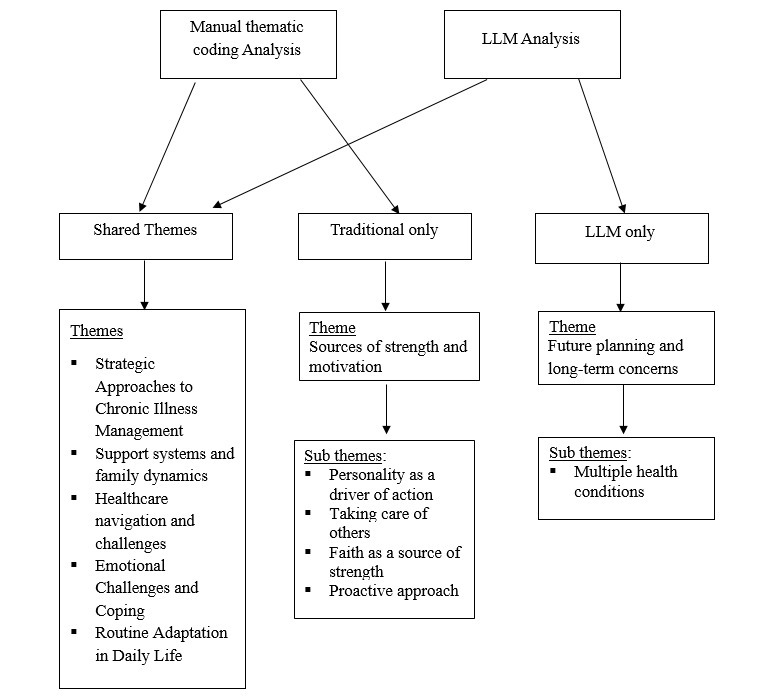
Comparison between manual thematic analysis and LLM-assisted analysis, showing shared themes and themes identified uniquely by each approach. Shared themes are presented without subthemes, as both methods produced similar patterns at that level, while subthemes are shown only for themes that differed between the approaches. LLM: large language model.

The traditional analysis identified 33 subthemes, while the LLM analysis uncovered 43 subthemes. Figure 2 presents a comparison between manual and LLM-assisted analyses, showing shared themes as well as themes identified uniquely by each approach. This figure visually summarizes the similarities and differences between the two analytical approaches. Many subthemes were consistent across both groups, with only 5 subthemes identified by the traditional analysis not appearing in the LLM analysis. Conversely, 1 subtheme was identified by the LLM analysis that was absent from the traditional analysis.

### Shared Themes

The comparative analysis revealed the following key findings identified by both methods. Illustrative quotes from participants appear in Tables 2 and 3.

### Strategic Approaches to Chronic Illness Management

This theme encompasses the strategies and efforts individuals use to manage and adapt to long-term health conditions. In the traditional analysis, illness management was described as an ongoing task embedded in daily routines. For example, patient 13 described actively managing his condition by monitoring and following up: “I don't give up on myself… I am writing to the doctor and ask for periodic tests. I have to keep an eye on my situation.” The LLM analysis reflected a similar focus, highlighting gradual acceptance and the integration of illness-related practices into one’s lifestyle. As patient 14 explained: “With awareness over the years, it really became part of my management… I learned to accept it.” More examples from participant narratives are presented in Tables 2 and 3.

### Support Systems and Family Dynamics

This theme explores how family relationships and external support networks influence individuals’ ability to manage chronic illness. It appeared as the role of support systems in the traditional analysis and as social and family dynamics in the LLM model. Both analyses emphasized the significant role of family and community in shaping patients’ experiences, ranging from emotional encouragement and practical help to tensions or ambivalence in relationships. The findings reflect the diverse scope of support, from sincere care to feelings of burden or intrusion. For example, in the traditional analysis, patient 2 described strong family and community support: “The community in the neighborhood where I live was very helpful… I learned to appreciate the power of family.” Similarly, the LLM analysis also reflected the importance of support, as seen in patient 13’s statement: “Without such support from the environment it's impossible… without my family, it's impossible.”

### Health Care Navigation and Challenges

This theme refers to the process of accessing and using health care services, and to the obstacles and frustrations patients face within the health care system. It was identified as health care and systemic challenges in the traditional analysis and as navigating health care systems in the LLM model. Participants described their journeys as bureaucratic, fragmented, and prolonged, often involving repeated referrals, inconsistent diagnoses, and delays before receiving effective care. These accounts reflect not only structural barriers but also a sense of exhaustion and uncertainty during the care-seeking process. For example, in the traditional analysis, patient 18 described the difficulty of receiving consistent diagnoses: “I went to about four dermatologists… each of them gave me a completely different diagnosis.” Similarly, the LLM analysis reflected the burden of repeated tests and long processes, as described by patient 20: “I had about a month filled with doing a million tests… I felt like I had been in the clinic for the whole month.”

### Emotional Challenges and Coping

This theme reflects the range of emotional responses individuals experience while managing chronic illness, including ongoing worry, frustration, guilt, and self-blame. Both traditional and LLM analyses highlighted how patients described emotional strain as part of daily life with chronic conditions. These expressions, while not always accompanied by explicit coping strategies, reveal an emotional labor patients engage in as they navigate uncertainty, treatment demands, and the psychological toll of illness. For example, in the traditional analysis, patient 6 described efforts to cope emotionally: “I try to protect my soul… with things that make me happy.” Similarly, the LLM analysis reflected emotional distress, as described by patient 14: “I had tears in my eyes… I feel like I failed.”

### Routine Adaptation in Daily Life

This theme captures the practical strategies and adjustments individuals make in their day-to-day lives to accommodate the demands of chronic illness. In the traditional analysis, it was labeled self-management and adaptation, while in the LLM model, it appeared as daily life and functional adjustments. Participants described changes to household routines, increased planning, and the gradual incorporation of external support when necessary. These narratives reflect a shift from full independence to negotiated interdependence, highlighting how people preserve autonomy while accepting limitations. For example, in the traditional analysis, patient 17 described changes in daily habits: “I have already changed my eating habits… I need to do some sport but I don't persist with it.” Similarly, the LLM analysis reflected adjustments in daily routines, as described by patient 2: “There are priorities… now with my eyes I'm not allowed to do many things so the helper comes twice a week.”

### Exclusive Themes

The comparative analysis revealed that certain themes emerged uniquely in one of the two analytic approaches: manual thematic analysis or LLM-assisted analysis. These differences reflect variation in how each method prioritized or interpreted aspects of participants’ narratives, rather than inconsistencies in accuracy.

### Sources of Strength and Motivation

This theme explores the factors that empower individuals to cope with challenges and pursue their goals. The traditional analysis uniquely identified this theme, highlighting both internal and external sources of motivation. Internal sources included personal faith, spiritual beliefs, and a proactive mindset that emphasized inner strength and optimism. External sources stemmed from caring responsibilities, encouragement from family members, and the need to maintain functional roles within the household.

These accounts illustrate how participants found motivation both within themselves and through their roles in the family and community. For example, patient 9 described a personal source of strength: “My strength comes from God… I believe in God.”

תחתית הטופס

### Future Planning and Long-Term Concerns

The LLM analysis uniquely identified this theme, which reflects participants’ concerns about the future and the strategies they use to prepare for potential long-term challenges. These included considerations such as financial stability, medical planning, and personal life goals. Although such reflections appeared in the traditional analysis as well, they were embedded within a broader subtheme of emotional challenges and coping, rather than being treated as a distinct theme. The LLM analysis, however, grouped these expressions, emphasizing their frequency and framing them as a separate area of concern regarding future planning. For example, patient 18 described concerns about the future: “I think about the diseases that I have… it can shorten a person's life.”

## Discussion

### Principal Findings

This study compared traditional qualitative thematic analysis with AI-based analysis using LLM to explore patients’ experiences in managing chronic illness. The analysis revealed 5 shared themes: strategic approaches to chronic illness management, support systems and family dynamics, health care navigation and challenges, emotional challenges and coping, and routine adaptation in daily life. In addition, each method revealed unique themes: the traditional analysis identified sources of strength and motivation, encompassing subthemes such as personality, caregiving roles, faith, and a proactive mindset. The LLM analysis uniquely identified future planning and long-term concerns, including issues related to multiple health conditions. These findings highlight the complementary roles of traditional and LLM-assisted analysis and contribute to the emerging methodological literature on AI-assisted qualitative research, which explores how LLMs can support, extend, and challenge traditional approaches to qualitative analysis. These findings are discussed in more detail in the following sections, focusing on both shared and unique themes.

### Themes Shared by The Two Qualitative Methods

The overlap between themes identified by the traditional and LLM-based analyses suggests that both approaches capture core aspects of chronic illness management, consistent with existing literature on chronic care and patient-centered management [25-27]. This indicates that, despite differences in the analytical process, both methods provide a meaningful understanding of the patient’s experience and highlight their complementary strengths. For example, health care navigation and challenges were a recurring theme in both approaches, with patients describing the bureaucratic hurdles and frustrations of accessing care. Studies on health care navigation for chronic patients highlight several common issues, such as fragmented care, inconsistent communication among providers, and a lack of standardized care pathways [28]. These challenges often result in delays in receiving accurate diagnoses and appropriate treatments, as patients navigate a complex health care system [29]. Furthermore, patients with multiple chronic conditions often receive conflicting medical advice and face barriers such as long wait times and limited access to care [30-33]. Together, these findings suggest that both analytical approaches can capture key systemic challenges experienced by patients with chronic illness.

The importance of support systems and family dynamics across both approaches aligns with previous research showing the central role of social support in chronic illness management [34,35]. Participants described the emotional, logistical, and practical support provided by family members, friends, and community networks, which helped them maintain a sense of balance and resilience amid the demands of their chronic conditions. The support systems and family dynamics theme aligns with research emphasizing the importance of family caregivers and social support in improving health outcomes and quality of life for individuals with chronic illnesses [36]. Social support has been shown to provide not only practical assistance but also a crucial psychological buffer against the stress and uncertainty of managing chronic conditions [37]. By fostering a strong support system, patients can enhance their overall quality of life and better cope with the physical and emotional challenges of living with chronic illness [38]. However, while social support is invaluable, it also comes with its complexities. Family dynamics may introduce challenges such as caregiver burnout, role strain, or feelings of dependence among patients [39,40], highlighting the importance of developing interventions that support both patients and caregivers [41,42]. Together, these findings suggest that both analytical approaches capture not only the benefits of social support but also its challenges, reflecting the complexity of patients’ lived experiences.

The emotional challenges and coping impact of chronic illness were evident across both analytical approaches, highlighting the central role of emotional challenges in patients’ experiences. Participants described the ongoing struggle to maintain a sense of normalcy and control amid the uncertainty of living with chronic conditions, as well as the impact on their mental health, including feelings of depression, anxiety, and grief over the loss of their previous way of life [36,43]. These findings align with existing research demonstrating the psychological burden associated with chronic illness, including an increased risk of mental health issues such as depression and anxiety, which can exacerbate physical symptoms and hinder effective self-management [36,44,45]. To cope, patients described a range of strategies, such as cultivating a positive mindset, engaging in self-care, and seeking support from professionals and peer networks [46,47]. Addressing these emotional needs is critical, as interventions targeting mental health can significantly improve overall health outcomes [48]. Together, these findings suggest that both analytical approaches capture not only the emotional burden of chronic illness but also the coping strategies patients use to manage these challenges.

Finally, chronic illness management, as reflected in both analytical approaches, encompasses a wide range of practices and experiences that can be viewed through both positive and challenging lenses. On the one hand, disease management can serve as a source of education and empowerment, enabling patients to take an active role in their care through self-monitoring and lifestyle adjustments, which may improve autonomy and health outcomes [49]. On the other hand, the burden of chronic illness management can be overwhelming, leading to burnout, reduced quality of life, and ongoing challenges in navigating the health care system [50]. Together, these findings highlight the complex and sometimes conflicting nature of chronic illness management, as experienced by patients. However, both analyses focused on identifying themes rather than applying a specific theoretical framework, and therefore remain mainly descriptive.

### Themes Exclusive to Only One Qualitative Method

The fact that the traditional analysis uniquely identified the theme of sources of strength and motivation suggests its strength in capturing more nuanced and deeply personal aspects of patient experience. This theme encompasses subthemes such as personality as a driver of action, taking care of others, faith as a source of strength, and a proactive approach. Participants described how personal beliefs, a sense of purpose, and relationships influenced their ability to manage their condition, such as patient 15’s connection to their professional identity. These findings are consistent with previous research showing that resilience, self-efficacy, and spiritual beliefs can play a central role in chronic illness management and are associated with improved coping and health outcomes [51-53]. Taking care of others can also strengthen patients’ ability to navigate care and maintain meaningful roles [54]. In addition, a positive mindset has been associated with better self-care behaviors and disease outcomes. These findings also reflect the role of personality traits, such as resilience and self-efficacy, in driving active engagement in illness management, supporting the subtheme of personality as a driver of action [52,55]. The subtheme of a proactive approach further reflects patients’ expectations for more active and supportive care. Participants emphasized the need for health care providers to take a more active role, such as scheduling follow-up appointments and maintaining ongoing engagement, rather than relying on patients to initiate contact. This finding aligns with existing literature on chronic disease management, which emphasizes the importance of planned and coordinated care that anticipates and addresses patient needs [49,56]. At the same time, this approach may not suit all patients, as some may perceive it as limiting their autonomy or creating additional pressure, highlighting the need to balance proactive care with respect for patients’ preferences and independence [57]. Together, these findings suggest that the traditional analysis can capture not only patient experiences, but also deeper, context-dependent aspects such as personal motivations, values, and expectations regarding care, which may be less evident in more structured analytical approaches.

The identification of multiple health conditions as a distinct subtheme highlights the ability of the LLM-assisted analysis to capture the complexity and accumulation of challenges faced by patients managing more than one chronic illness. This subtheme reflects the combined burden of multiple conditions, each with its own symptoms, treatments, and impact on daily life. As described by patient 7, “It's not just one thing, it's everything together. I have diabetes, high blood pressure, arthritis, and now this new thing with my heart. It's just too much sometimes.” Managing multiple chronic illnesses often requires navigating a fragmented health care system, coordinating care among multiple providers, and following complex and sometimes conflicting treatment plans [58]. Patients may also experience a compounding effect, where one condition exacerbates the symptoms or management challenges of another, increasing the overall burden and complexity of care [59]. These findings suggest that LLM-assisted analysis may be particularly effective in identifying patterns related to complexity, accumulation, and co-occurring conditions within patient narratives. By highlighting these patterns, this approach may support a more system-level understanding of chronic illness management.

Overall, this study highlights the complementary value of traditional and LLM-assisted qualitative analysis in understanding chronic illness management. While traditional analysis provided deeper insight into personal meanings, motivations, and patient perspectives, the LLM-assisted approach was effective in identifying broader patterns and capturing the complexity of managing multiple conditions. Together, these findings suggest that integrating both approaches can enhance the depth, scope, and applicability of qualitative health research, particularly in complex clinical contexts.

### Study Limitations and Future Directions

This study provides a valuable comparison between traditional qualitative analysis and AI-based analysis using Claude 3.5 Sonnet. However, this reliance on a single LLM constitutes a key limitation, as it does not account for potential variations in performance and insights that may arise from using other LLMs, such as ChatGPT, Gemini (Google LLC), or other emerging systems. Future studies should explore a comparative evaluation across multiple LLMs to better understand the range of capabilities, biases, and thematic coverage. Another limitation relates to the dataset and its contextual dependency. While the dataset was sufficient for this analysis, its characteristics may have influenced the themes identified by both methods. Broader and more diverse datasets could support more comprehensive comparisons. Participant recruitment through primary care physicians may have introduced selection bias, as individuals who are more communicative or more engaged with the health care system may have been more likely to be referred to. This may have influenced the range of perspectives represented in the sample. The comparison between the two approaches was based on predefined qualitative criteria and remained primarily interpretive and descriptive, without formal quantitative evaluation. In addition, the traditional qualitative analysis relied on a single researcher, which may have increased the risk of interpretive bias, although reflexive practices were used to mitigate this. Another limitation is that both the manual and LLM analyses were not theory-driven, resulting in findings that are primarily descriptive rather than theoretically grounded. Future research should explore how theoretical frameworks can be integrated into both manual and LLM-assisted analysis. Similarly, while LLMs offer scalability and efficiency, their output depends on the training data, which introduces biases or overlooks important contextual nuances. Moreover, the use of an LLM raises ethical considerations, including transparency, interpretability, and the potential for misrepresentation of participants’ voices. In this study, the model operated solely on anonymized transcripts and was used to support, rather than replace, human interpretation. Finally, this study did not assess the reliability of LLM-generated themes or their clinical applicability to real-world scenarios. Therefore, these findings should be interpreted as exploratory thematic observations rather than clinically validated psychosocial classifications. Future research should integrate mixed-method approaches to evaluate the validity, reproducibility, and practical relevance of themes generated by LLMs. More broadly, future studies should aim to integrate AI-driven tools into qualitative research designs in ways that enhance both efficiency and analytical depth. The development of hybrid frameworks that use LLM-based pattern recognition with human-led contextual interpretation represents a promising direction. Additionally, longitudinal research and real-world applications will be important for assessing the stability and clinical relevance of LLM-generated insights.

### Conclusions

This study examined the use of traditional qualitative thematic analysis and LLM-based analysis to explore patients’ experiences in managing chronic illness. Both approaches identified similar core themes, while also revealing distinct insights.

These findings suggest that the two approaches offer complementary perspectives. Traditional analysis enabled a rich, interpretive understanding of patient narratives, while the LLM approach supported efficient and systematic identification of thematic structures. While AI-based methods hold potential to support qualitative research—particularly in scaling and organizing data—they require careful validation to ensure cultural and contextual sensitivity. Taken together, this study highlights the value of a hybrid approach, integrating both human insight and AI capabilities, to enhance the depth and scope of qualitative health research.

## Data Availability

The datasets used and/or analyzed during this study are available from the corresponding author on reasonable request.

## Abbreviations

AI: artificial intelligence
LLM: large language model
